# Rifampin-resistant *Neisseria meningitidis*

**DOI:** 10.3201/eid1205.051296

**Published:** 2006-05

**Authors:** Muhamed-Kheir Taha, Maria Leticia Zarantonelli, Corinne Ruckly, Dario Giorgini, Jean-Michel Alonso

**Affiliations:** * Institut Pasteur, Paris, France

**Keywords:** resistance, virulence, meningitis, animal model, letter

**To the Editor:** Immediate management of meningococcal disease requires antimicrobial drug treatment of patients with β-lactams and chemoprophylaxis of contact persons with rifampin. High-level resistance to rifampin (MIC >32 mg/L) in *Neisseria meningitidis* is provoked by mutations (most frequently at the residue His 552) in the *rpoB* gene encoding the b subunit of RNA polymerase (*1**,**2*). Resistance may lead to chemoprophylaxis failure and must be rapidly detected (*3*). Concerns have been raised about the clonal spread of resistant isolates (*1*); however, rifampin-resistant isolates are rarely reported. We tested 6 *N. meningitidis* isolates corresponding to 3 pairs of linked cases of meningococcal disease. In each pair, the index case was due to a rifampin-susceptible isolate and was followed by the secondary case due to a resistant isolate in a contact person. Phenotyping and genotyping of the isolates showed that each pair belonged to a different major serogroup (A, B, and C) and to a different genetic lineage (ST-7, ST-32, and ST-2794) (Figure). We next amplified a fragment in *rpoB* between codons 421 and 701 by using oligonucleotide rpoBF1 (5´gttttcccagtcacgacgttgtaCTGTCCGAAGCCCAACAAAACTCTTGG3´) and rpoBR1 (5´ttgtgagcggataacaatttcTTCCAAGAATGGAATCAGGGATGCTGC3´). The 2 oligonucleotides harbor adaptors (in lower case) corresponding to universal forward and reverse oligonucleotides that can be used for sequencing after amplification. We also analyzed 2 cerebrospinal fluid (CSF) samples corresponding to 2 linked culture-negative cases of meningococcal disease in which the second case was believed to have been caused by rifampin-resistant *N. meningitidis*. These 2 cases were diagnosed by polymerase chain reaction (PCR) detection of meningococcal DNA, as previously described (*4*).

**Figure:**
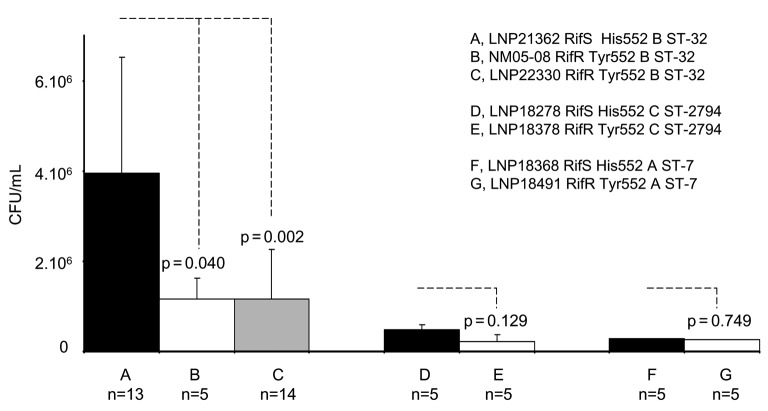
Blood bacterial counts in 6-week-old female BALB/c mice (Janvier, France), challenged intraperitoneally with standardized inocula of 10^7^ colony forming units (CFU) of rifampin-susceptible (RifS) isolates and their corresponding rifampin-resistant (RifR) isolates. Bacteremia was followed at 2 and 4 h after challenge. Only results after 4 h of challenge are shown. The name of the isolates tested, their phenotype (susceptibility to rifampin, RifS or RifR, residue at the position 552 and serogroups B, C, and A), and their genotype (sequence type ST) are indicated. Results are the means ± standard error (bars) from groups of at least 5 mice (the number of mice, n, is given above each histogram). p values were determined by 2-tailed Student *t* test.

The 3 rifampin-susceptible isolates harbored a wild-type *rpoB* sequence (His 552), as did the first CSF sample. All 3 rifampin-resistant isolates harbored a His→Tyr mutation, while analysis of the second CSF sample showed a His→Asn mutation (Figure). Both mutations have been observed in *N. meningitidis* (*3*). No other difference in the sequence was seen among all isolates on the amplified fragment. This approach can rapidly detect *rpoB* mutations and can be applied to culture-negative clinical samples.

The virulence of the isolates was evaluated through their ability to provoke bacteremia in mice after 6-week-old female BALB/c mice (Janvier, France) were injected intraperitoneally. Bacteremia is a good indicator of bacterial virulence as it reflects bacterial survival upon invasion of the bloodstream. The experimental design was approved by the Institut Pasteur Review Board. The rifampin-resistant clinical isolate LNP22330 showed substantially reduced bacteremia when compared to the corresponding susceptible isolate LNP21362 (Figure). Such a reduction was not significant for the other 2 pairs (LNP18278/LNP18378 and LNP18368/LNP18491), but these strains were all less virulent than LNP21362, with ≈1 log_10_ lower blood bacterial loads. The 3 pairs of isolates belonged to different genetic lineages according to the multilocus sequence typing typing. Indeed, we have recently proved that virulence of meningococcal isolates in the mouse model depends on the genetic lineage of the tested isolate (*5*).

To better study the impact of *rpoB* mutation on meningococcal virulence we constructed an isogenic mutant strain, NM05-08, by transforming the susceptible isolate LNP21362 with a PCR-amplified fragment from a resistant isolate (LNP22330), as previously described (*6*). The PCR fragment corresponded to the product of amplification between the oligonucleotides ropB1UP (5´ggccgtctgaaCTGTCCGAAGCCCAACAAAACTCTTGG3´) and rpoBR1. The oligonucleotide RpoB1UP is the same as the upstream rpoBF1 but with a DNA uptake sequence (in lower case) that was added at the 5´ end to permit DNA transformation (*7*). The transformant strain NM05-08 was resistant to rifampin (MIC >32 mg/L), and the sequence of the *rpoB* gene confirmed the His→Tyr mutation. When compared to the parental isolate (LNP21362), strain NM05-08 showed reduced virulence. Indeed, bacterial loads were similar to those observed for the resistant isolate LNP22330 (Figure). These results strongly suggest a direct negative impact of *rpoB* mutations on meningococcal virulence. Mutations in the *rpoB* gene have been reported to confer pleiotropic phenotypes (*8*).

The data reported here show that rifampin-resistant isolates were not clonal but belonged to different genetic lineages. The results of virulence assays in mice suggest that mutations in *rpoB* in resistant isolates may have a major biological cost for *N. meningitidis*, which can be defined as lower bacterial fitness in terms of survival in the bloodstream. This biological cost could explain the lack of clonal expansion of meningococcal isolates that acquired resistance to rifampin.
